# TFANet: a temporal fusion attention neural network for motor imagery decoding

**DOI:** 10.3389/fnins.2025.1635588

**Published:** 2025-09-22

**Authors:** Chao Zhang, Ya Liu, Xiaopei Wu

**Affiliations:** Anhui University Laboratory of Intelligent Information and Human-Computer Interaction, Anhui University, Hefei, China

**Keywords:** brain-computer interface, motor imagery, electroencephalogram, temporal dependencies, multi-scale temporal self-attention, temporal depthwise separable convolutional fusion network

## Abstract

**Introduction:**

In the field of brain-computer interfaces (BCI), motor imagery (MI) classification is a critically important task, with the primary objective of decoding an individual's MI intentions from electroencephalogram (EEG) signals. However, MI decoding faces significant challenges, primarily due to the inherent complex temporal dependencies of EEG signals.

**Methods:**

This paper proposes a temporal fusion attention network (TFANet), which aims to improve the decoding performance of MI tasks by accurately modeling the temporal dependencies in EEG signals. TFANet introduces a multi-scale temporal self-attention (MSTSA) mechanism that captures temporal variation in EEG signals across different time scales, enabling the model to capture both local and global features. Moreover, the model adaptively adjusts the channel weights through a channel attention module, allowing it to focus on key signals related to motor imagery. This further enhances the utilization of temporal features. Moreover, by integrating the temporal depthwise separable convolution fusion network (TDSCFN) module, TFANet reduces computational burden while enhancing the ability to capture temporal patterns.

**Results:**

The proposed method achieves a within-subject classification accuracy of 84.92% and 88.41% on the BCIC-IV-2a and BCIC-IV-2b datasets, respectively. Furthermore, using a transfer learning approach on the BCIC-IV-2a dataset, a cross-subject classification accuracy of 77.2% is attained.

**Conclusion:**

These results demonstrate that TFANet is an effective approach for decoding MI tasks with complex temporal dependencies.

## 1 Introduction

Brain-computer interfaces (BCI) technology allows the human mind to directly connect with external systems, enabling novel forms of interaction (Wolpaw et al., 2002). Motor imagery (MI) based on electroencephalography (EEG) has emerged as a prominent and widely studied paradigm in brain-computer interface research. In BCI research, this methodology has demonstrated cross-disciplinary applicability, with particularly transformative impacts in medical applications (Altaheri et al., 2023). However, the limited robustness against noisy EEG signals and the inherent variability of brain activity pose significant challenges to the accurate interpretation of neural signals (Hsu and Cheng, 2023). These factors can lead to inconsistent output results, thereby compromising the reliability and efficacy of MI-EEG decoding.

Traditional machine learning generally consists of two stages: feature extraction and classifier design. Common feature extraction methods include various techniques, such as wavelet transform (WT) (Zhang and Zhang, 2019), which decomposes signals into time-frequency representations and enables analysis of signal characteristics within specific time intervals and frequency bands; principal component analysis (PCA) (Abdi and Williams, 2010), which extracts the main directions of variance in the data through dimensionality reduction and is often used to remove redundant information; and common spatial pattern (CSP) (Ramoser et al., 2000), which improves classification accuracy by identifying spatial features associated with different tasks or states. Based on CSP, numerous variants have been developed to enhance decoding performance, such as regularized common spatial pattern (RCSP) (Lotte and Guan, 2010) and filter bank common spatial pattern (FBCSP) (Ang et al., 2008). After feature extraction, feature classification is typically performed, using machine learning algorithms to identify user intent. Commonly used classification algorithms include k-nearest neighbors (KNN) (Peterson, 2009), which classify data by calculating the distance between input samples and training set samples; support vector machines (SVM) (Hearst et al., 1998), which separate data into different classes by finding an optimal hyperplane; linear discriminant analysis (LDA) (Balakrishnama and Ganapathiraju, 1998), which effectively reduces dimensionality for classification, and naive Bayes (NB) (Murphy, 2006) classifier categorizes data by calculating the posterior probability for each class. Although these methods perform well in MI-EEG decoding, most still rely heavily on handcrafted features.

The decoding problem of MI has become a key limiting factor hindering the further development of the MI-BCI field (Craik et al., 2019). With ongoing advancements in computer science, deep learning (DL) (LeCun et al., 2015) has increasingly been utilized in the development of decoding algorithms, with convolutional neural networks (CNNs) (Li et al., 2021) being the most popular among them. However, their fixed receptive field limits their performance on time-series data, making it difficult to effectively capture long-duration temporal dependencies. To address this, a temporal convolutional network (TCN) (Bai et al., 2018) based on CNNs has been proposed, focusing on time-series modeling and classification. In comparison, recurrent neural networks (RNNs) (Sherstinsky, 2020) are more susceptible to issues like vanishing or exploding gradients. Compared to RNN-based methods such as gate recurrent unit (GRU) (Chung et al., 2014) and long short-term Memory (LSTM) (Graves, 2012), TCNs have demonstrated superior performance in time-series tasks. ETCNet (Qin et al., 2024) combines efficient channel attention (ECA) (Wang et al., 2020) and TCN components to extract channel features and temporal information. EEG-TCNet (Ingolfsson et al., 2020) combines EEGNet with TCN, enabling more effective processing and analysis of time series data. TCNet-Fusion (Musallam et al., 2021) builds upon EEG-TCNet by adding layer fusion, reducing feature loss, and constructing rich feature mappings.

In recent years, researchers have discovered unexpected advantages in integrating attention mechanisms into deep learning models. The attention mechanism (Vaswani, 2017) simulates the human process of selective information focus, enabling models to concentrate on important elements while ignoring irrelevant content. These mechanisms mimic human perception patterns and attention behavior, allowing neural networks to distinguish between key information and secondary data. The Multi-Head Attention mechanism (MHA) (Vaswani, 2017) enables the parallel processing of various global temporal features. In this context, ATCNet (Altaheri et al., 2022) uses MHA to highlight key information in EEG time series signals. Conformer (Song et al., 2022) uses the MHA module to capture global long-term dependencies on top of the local temporal features extracted by CNN. TMSA-Net (Zhao and Zhu, 2025) integrates dual-scale CNNs with the attention mechanism in MHA modules, effectively capturing global dependencies. MSCFormer (Zhao et al., 2025) combines multi-branch CNNs and MHA modules to address individual variability in EEG signals. These methods dynamically assign higher weights to task-relevant temporal segments in the input sequence, thereby highlighting discriminative EEG patterns. However, existing attention mechanisms typically focus on dependencies at a single time scale, while EEG signals exhibit multi-scale dependencies in the time domain. Existing models struggle to simultaneously capture long- and short-term, multi-scale temporal dependencies.

Based on the aforementioned challenges, this paper proposes an innovative end-to-end deep learning architecture. This network is capable of accurately modeling the temporal dependencies of EEG signals, thereby enhancing decoding performance. First, the convolution extracts low-level features. Second, an attention module is used to more effectively extract and fuse features, highlighting the most important parts of the time series. Finally, improved TCN extracts high-level temporal features.

The contributions of the proposed TFANet model can be summarized as follows:

1. A multi-scale temporal self-attention (MSTSA) module is designed, integrating multi-scale temporal convolutional blocks and self-attention blocks. This module can simultaneously capture both local and global features while dynamically adjusting its focus on critical information.

2. By combining the SE module with the MSTSA module, an innovative spatio-temporal attention fusion is achieved. This approach enhances the focus on temporal scales while also improving the specificity of channel weights.

3. The TCN module has been improved by replacing the expanded causal convolution with expanded causal depthwise separable convolution in the first residual block. In the second residual block, the residual connection of the TCN was modified to a multi-level residual connection. These adjustments not only maintain a low parameter count but also achieve multi-level feature fusion.

4. To address the inherent challenge of limited EEG trial samples in standard BCI datasets (due to clinical trial limitations, there are 288 trials/subjects in the BCI-IV-2a dataset), we developed a novel temporal segmentation and recombination augmentation strategy. This approach divides each trial into 8 physiologically meaningful segments and systematically recombines them within the same class, thereby significantly expanding training dataset diversity while maintaining the integrity of task-relevant neural patterns.

The structure of this paper is as follows: Section 2 describes the proposed model; Section 3 details the experimental setup and discusses the results; Section 4 provides the conclusion.

## 2 Materials and methods

### 2.1 Datasets

This study utilized the two most widely used public datasets in the field of MI classification for evaluation: BCI Competition IV 2a (Brunner et al., 2008) and BCI Competition IV 2b (Leeb et al., 2008).

#### 2.1.1 BCIC-IV-2a

The BCIC-IV-2a dataset records four-class MI tasks (left hand, right hand, both feet, and tongue) performed by 9 subjects, with data collected from 22 channels at a sampling rate of 250 Hz. For each subject, two separate sessions were recorded on different days: the data from the first session were used for model training, while the data from the second session were reserved for model testing. Each session consists of 288 trials, with 72 trials per MI task. In this experiment, the onset of the visual cue served as the temporal anchor point (*t* = 0). Samples were extracted from EEG segments within the (0,4) second window following the visual cue onset (corresponding to the absolute time window of 2–6 s after trial initiation, since the cue appeared 2 s into the trial in the BCIC-IV-2a dataset), yielding 4 s of data. At a sampling rate of 250 Hz, this resulted in 1,000 time points per sample.

#### 2.1.2 BCIC-IV-2b

The BCIC-IV-2b dataset records EEG signals from 9 subjects performing two-class MI tasks (left hand and right hand) using three electrode channels (C3, Cz, C4) sampled at 250 Hz. Each subject completed five recording sessions across different days, with the first two sessions containing 120 feedback-free trials each and the subsequent three sessions comprising 160 online feedback trials each. For experimental purposes, the first three sessions (totaling 400 trials) were used for training while the remaining two sessions (totaling 320 trials) served as the test set. In this experiment, the onset of the visual cue served as the temporal anchor point (*t* = 0). Samples were extracted from EEG segments within the (0,4) second window following the visual cue onset (corresponding to the absolute time window of 3–7 s after trial initiation, since the cue appeared 3 s into the trial in the BCIC-IV-2b dataset), yielding 4 s of data. At a sampling rate of 250 Hz, this resulted in 1,000 time points per sample.

### 2.2 Input representation and preprocessing

The EEG signals used in this study were obtained from the publicly available BCIC-IV-2a and BCIC-IV-2b datasets in their originally distributed form. These datasets were preprocessed by the providers with standard techniques prior to release, including: A band-pass filter (0.5–100 Hz) to remove extremely low and high frequency artifacts and a 50 Hz notch filter to eliminate power line interference.

### 2.3 Data augmentation

To address the limited quantity of EEG trials and mitigate class imbalance, this paper proposes a data augmentation method based on temporal segmentation and recombination of time series. This technique divides each multi-channel EEG trial into 8 non-overlapping temporal segments (segment length = step size = 125 time points), while preserving the original channel groupings to maintain spatial correlations. During the random recombination of segments from the same class, these segments are concatenated to generate new samples. This process preserves the consistency of class-specific features while introducing data diversity. This augmentation is exclusively applied to the training set, while test data remains strictly unaugmented. Crucially, test data is completely isolated from the augmented training set. This method performs well on time series datasets, effectively improving the model's generalization ability in scenarios where class samples are scarce or data distribution is imbalanced.

### 2.4 Proposed TFANet architecture

**Figure 1:**
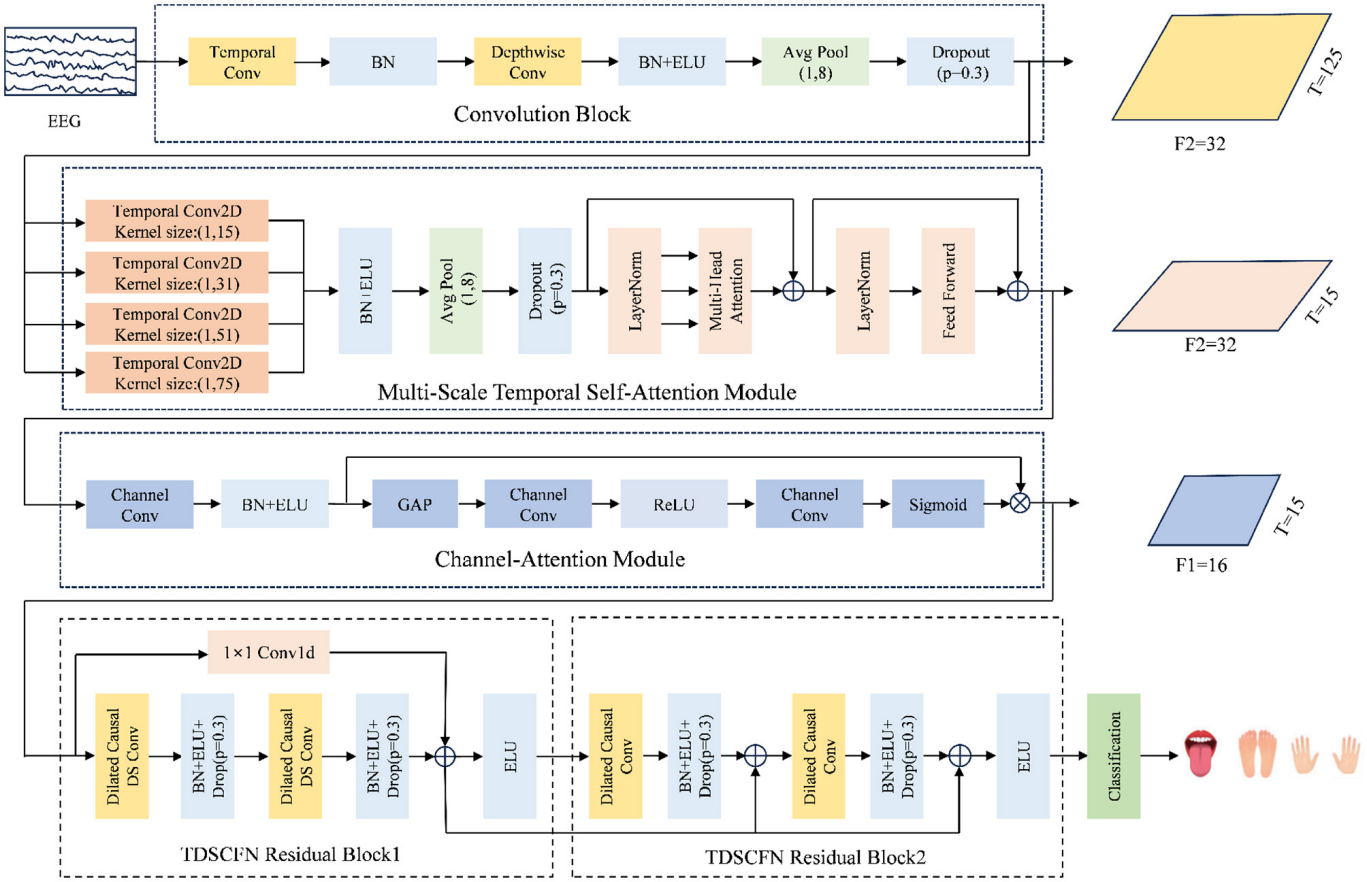
The architecture of TFANet consists of four key components: a convolutional block, a multi-scale temporal self-attention (MSTSA) module, an Squeeze-and-Excitation (SE) module, and a temporal depthwise separable convolution fusion network (TDSCFN) module.

The framework of TFANet is shown in Figure 1. The model consists of convolutional blocks, the MSTSA module, the SE module, and the temporal depthwise separable convolution fusion network (TDSCFN) module.

First, the augmented EEG signals are processed through a convolutional block containing temporal and spatial filters to extract preliminary temporal features. This step captures spatiotemporal information in the signal through local convolution operations, providing a foundation for subsequent temporal modeling. The MSTSA module allows the model to apply attention weighting across different temporal scales, enabling it to focus on both local and global features. A channel compression module is then added to reduce computational complexity and parameter size, while further processing and optimizing the features. The SE module further enhances feature selectivity, helping the model focus on key moments within the global context. Subsequently, the introduction of TDSCFN enables the model to handle advanced temporal features. The final step of the classification process is to pass the extracted features to the fully connected (FC) layer.

### 2.5 Convolution block

The convolutional module is comprised of a temporal and a spatial filter. Initially, the EEG signal is processed by the temporal filter, which is structured with a two-dimensional convolutional layer and a batch normalization (BN) (Santurkar et al., 2018) layer. This assembly can extract temporal characteristics across varied frequency bands through the application of a convolutional kernel (*F*_1_ = 16) characterized by a kernel dimension of (1, 32). The second layer employs channel convolution, utilizing depthwise convolution with *F*_2_ convolution kernels sized (C, 1) and groups set to *F*_1_, to learn spatial filters specific to each band. The variable C represents the number of channels, while *F*_2_ indicates the dimensionality of the output features from the convolution block. The value of *F*_2_ is computed as *F*_2_ = D × *F*_1_, Where D represents the connectivity degree between the preceding and the current layer, which is empirically determined to be 2. Subsequently, a BN layer and the exponential linear unit (ELU) (Clevert, 2015) activation function are applied to enhance the model's generalization ability and nonlinear expressive power. Next, by applying an average pooling operation with a kernel size of (1, 8), the temporal dimension of the input is effectively reduced. This not only decreases the number of parameters but also enhances computational efficiency. Dropout regularization is also applied to prevent overfitting (Salehin and Kang, 2023). Table 1 provides the specific parameters for constructing the convolution block.

**Table 1:** Architecture specification of the convblock module in TFANet.

**Layer**	**Filters**	**Size**	**Stride**	**Padding**	**Activation**	**Out**	**Parameters**
Input	–	–	–	–	–	(1, *C, T*)	0
Conv2D	*F*_1_	(1,32)	(1,1)	(0,16)	–	(*F*_1_, *C, T*)	512
BatchNorm2D	–	–	–	–	–	(*F*_1_, *C, T*)	32
DepthwiseConv2D	*D*×*F*_1_	(C,1)	(1,1)	0	–	(*F*_2_, 1, *T*)	704
BatchNorm2D	–	–	–	–	–	(*F*_2_, 1, *T*)	64
Activation	–	–	–	–	ELU	(*F*_2_, 1, *T*)	0
AvgPool2D	–	(1,8)	(1,8)	–	–	(*F*_2_, 1, *T*/8)	0
Dropout = 0.3	–	–	–	–	–	(*F*_2_, 1, *T*/8)	0
Total							1,312

### 2.6 Attention module

#### 2.6.1 Multi-scale temporal self-attention mechanism

In the BCI-MI decoding process, convolutional neural networks with a single scale suffer from limited receptive fields, which leads to inadequate feature extraction. This limitation hinders the effective perception and capture of global dependencies in EEG signals, thus restricting the model's performance when handling complex and global features. To address this, the MSTSA designed in this paper effectively captures key features of EEG signals at different time scales, overcoming the limitations of CNNs in modeling temporal information, especially the challenges posed by inter-individual differences in EEG signals. By adaptively focusing on important information across different scales and time periods, it overcomes the shortcomings of single-scale feature extraction.

**Figure 2:**
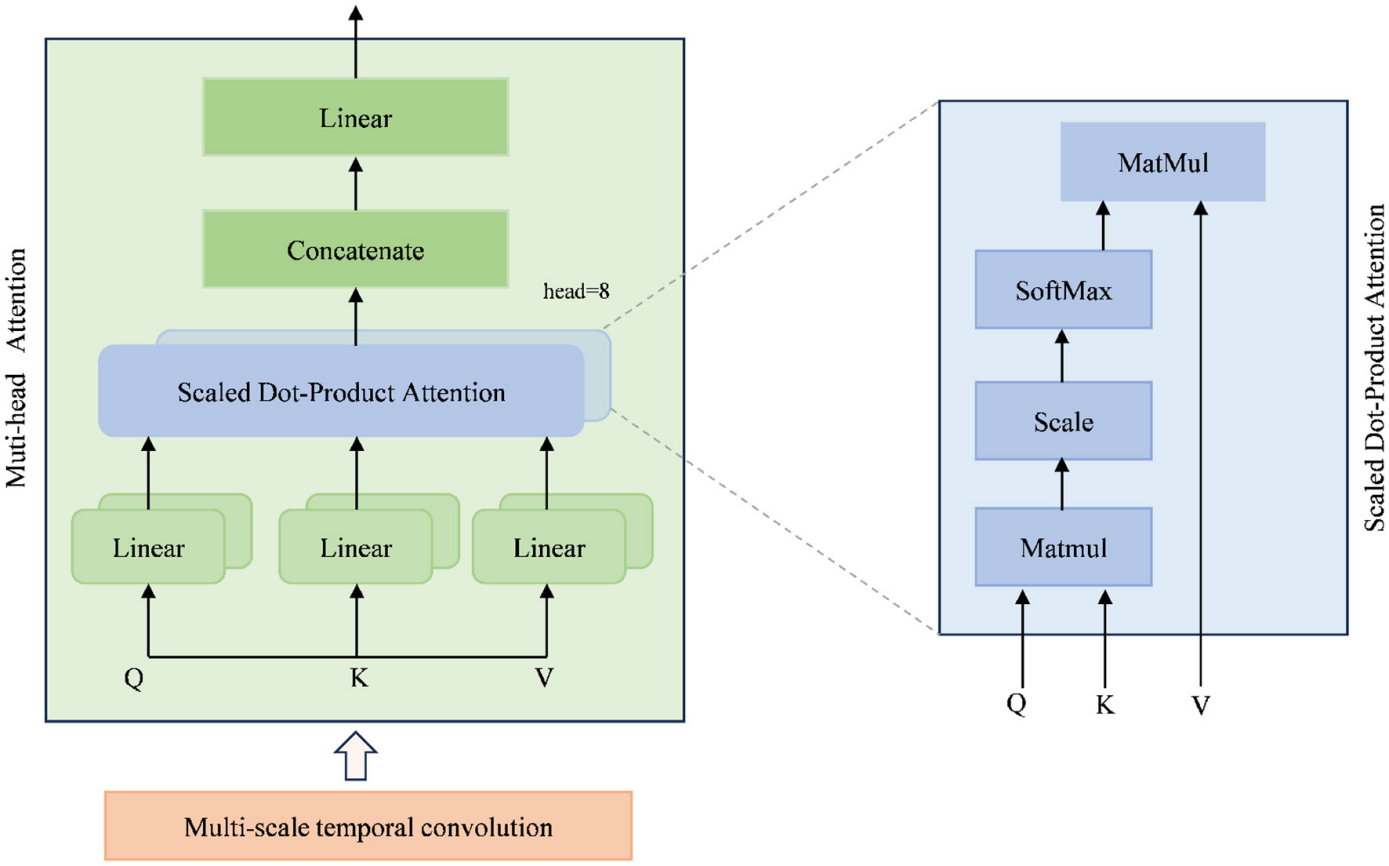
Multi-scale temporal self-attention mechanism.

The MSTSA module consists of multi-scale temporal convolutions and a self-attention (SA) module. The multi-scale temporal convolution applies a group of temporal filters with four 2D convolution layers, having kernel sizes of (1, 15), (1, 31), (1, 51), and (1, 75), respectively, to extract local temporal information. In Figure 2, the multi-scale temporal convolution, compared to a single temporal filter, is capable of extracting features at different time scales. The quartet of outputs from the temporal filter ensemble is then concatenated along the convolutional feature channel axis. Then, BN and the ELU activation function are applied along the feature map dimension, followed by further average pooling. The specific parameters for constructing the multi-scale convolution block are provided in Table 2.

**Table 2:** Architecture specification of the multi-scale temporal convolution module in TFANet.

**Layer**	**Filters**	**Size**	**Stride**	**Padding**	**Activation**	**Out**	**Parameters**
Input	–	–	–	–	–	(*F*_2_, 1, *T*/8)	0
TempConv1	*F*_2_/4	(1, 15)	(1, 1)	(0, 7)	–	(*F*_2_/4, 1, *T*/8)	3,848
TempConv2	*F*_2_/4	(1, 31)	(1, 1)	(0, 15)	–	(*F*_2_/4, 1, *T*/8)	7,944
TempConv3	*F*_2_/4	(1, 51)	(1, 1)	(0, 25)	–	(*F*_2_/4, 1, *T*/8)	13,064
TempConv4	*F*_2_/4	(1, 75)	(1, 1)	(0, 37)	–	(*F*_2_/4, 1, *T*/8)	19,208
Concat	–	–	–	–	–	(*F*_2_, 1, *T*/8)	0
BatchNorm2D	–	–	–	–	–	(*F*_2_, 1, *T*/8)	64
Activation	–	–	–	–	ELU	(*F*_2_, 1, *T*/8)	0
Shape	–	–	–	–	–	(*F*_2_, *T*/8)	0
Dropout = 0.3	–	–	–	–	–	(*F*_2_, *T*/64)	0
AvgPool1D	–	8	8	–	–	(*F*_2_, *T*/64)	0
Total							44,128

The self-attention (SA) module consists of two parts. The first part is the MHA, which can be described as:


(1) $$\begin{array}{c} Attention\left(Q,K,V\right)=softmax\left(\frac{QK^{T}}{\sqrt{d_{k}}}\right)V \end{array}$$

Queries (Q), Keys (K), and Values (V) are matrices composed of vectors for parallel processing. The parameter *d*_*k*_ represents the dimension of each head. MHA learns different features of the input data in parallel through multiple independent attention heads. The attention process can be expressed as:


(2) $$\begin{array}{l} MHA(Q,K,V)=\text{Concat}(head_{1},\ldots ,head_{k})W^{O}\\ \;head_{i}=\text{Attention}(QW_{i}^{Q},KW_{i}^{K},VW_{i}^{V})\\ \;[P_{MHA}=4\times d\times (d+1)=4\times 32\times (32+1)=4224] \end{array}$$

where *head*_*i*_ is the output of the i th, $W_{i}^{Q}\in {\mathbb{R}}^{d\times d_{q}}$, $W_{i}^{K}\in {\mathbb{R}}^{d\times d_{k}}$, $W_{i}^{V}\in {\mathbb{R}}^{d\times d_{v}}$, $W_{i}^{O}\in {\mathbb{R}}^{hd_{v}\times d}$, $d_{q}=d_{k}=d_{v}=\frac{d}{h}=\frac{32}{8}=4$.

The second layer consists of linear transformations followed by a GELU (Hendrycks and Gimpel, 2016) activation function. Additionally, layer normalization (Ba, 2016) and residual connections (He et al., 2016) are introduced.

#### 2.6.2 Channel attention

Since the brain functional areas corresponding to different body parts vary when performing different motor imagery tasks (Mnih et al., 2014), treating all channels equally may fail to give more attention to channels highly related to the motor imagery task. This could negatively impact the quality of spatial feature extraction, ultimately leading to poor classification performance. In the model, the SE module is incorporated to dynamically weight each channel, enhancing the representation of important features and reducing the interference from irrelevant ones. The model not only enhances its expressive capability along the channel dimension but also works synergistically with other time-series modules to leverage their combined strengths. First, the input feature $X_{c}\in {\mathbb{R}}^{F\times C\times T}$ is compressed into a feature vector through global average pooling, where F, C, and T denote the quantities of feature maps, channels, and sampling points, respectively. As follows:


(3) $$\begin{array}{c} z_{c}=\frac{1}{C\times T}\overset{C}{\underset{j=1}{\sum }}\overset{T}{\underset{j=1}{\sum }}X_{c}\left(i,j\right),\;c=1,2,\ldots ,F \end{array}$$

Subsequently, two FC layers are employed to capture the intricate nonlinear dependencies among the various feature representations. The process can be expressed as:


(4) $$\begin{array}{l} W=\sigma (W_{2}\delta (W_{1}Z))\;[P_{W}=\frac{F}{r}\times F+F\times \frac{F}{r}\\ \;=4\times 16+16\times 4=128,r=4] \end{array}$$

Specifically, W represents the weights, where $W_{1}\in {\mathbb{R}}^{\frac{F}{r}\times F}$ is the weight matrix of the initial FC layer, and $W_{2}\in {\mathbb{R}}^{F\times \frac{F}{r}}$ is the weight matrix of the subsequent FC layer that restores the features to their original dimensions. ReLU (Agarap, 2018) and sigmoid (Han and Moraga, 1995) activation functions are denoted by δ and σ, respectively.

Additionally, a 1 × 1 convolution is performed between MSTSA and SE to reduce the input feature channel dimension from *F*_2_ to *F*_1_. This adjustment in the number of channels helps reduce computational cost, control the model size, and facilitates further feature extraction and processing. The specific parameters of the channel module are shown in Table 3.

**Table 3:** Architecture specification of the channel processing module in TFANet.

**Layer**	**Filters**	**Size**	**Stride**	**Padding**	**Activation**	**Out**	**Parameters**
Input	–	–	–	–	–	(*F*_2_, 1, *T*/8)	0
Conv2D	*F*_1_	(1, 1)	(1, 1)	0	–	(*F*_1_, 1, *T*/8)	512
BatchNorm2D	–	–	–	–	–	(*F*_1_, 1, *T*/8)	32
ELU	–	–	–	–	ELU	(*F*_1_, 1, *T*/8)	0
AdaptiveAvgPool2D	–	–	–	–	–	(*F*_1_, 1, 1)	0
Conv2D (FC1)	*F*_1_/4	(1, 1)	(1, 1)	0	ReLU	(*F*_1_/4, 1, 1)	64
Conv2D (FC2)	*F*_1_	(1, 1)	(1, 1)	0	Sigmoid	(*F*_1_, 1, 1)	64
Scale multiply	–	–	–	–	–	(*F*_1_, 1, *T*/8)	0
Total							672

### 2.7 Temporal depthwise separable convolutional fusion network

**Figure 3:**
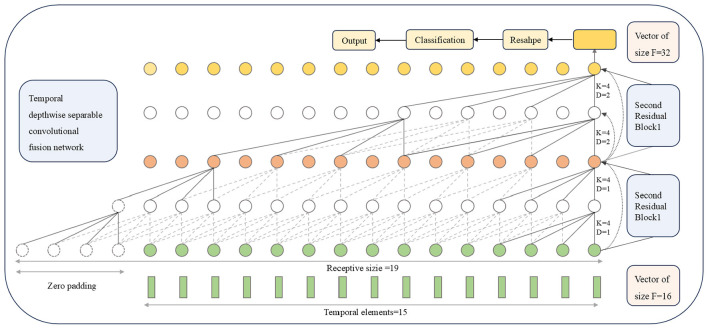
Visualization of the feature map of the TDSCFN module based on 15 time elements.

The design of the TDSCFN model is similar to the TCN network proposed in Bai et al. (2018). As show in Figure 3. To streamline the module's parameter count, an expanded causal depthwise separable convolution is used in the first residual unit, replacing the original expanded causal convolution, while preserving its decoding performance. This improvement includes a layer of dilated causal depthwise convolution and a layer of pointwise convolution. In the second residual block, the fusion block in the TDSCFN module replaces the TCN residual block, substituting the original residual connection with a multi-level residual connection, achieving multi-level feature fusion, which enriches the feature information while alleviating model overfitting.

The dilated convolution employed in this architecture employs an exponentially increasing dilation factor across the causal depth. Specifically, for the i th residual block, the dilation factor is defined as 2^*i*^−1. The exponential advancements in dilation effectively expand the temporal receptive field without a proportional increase in computational complexity. Its receptive field size (RFS) is defined as


(5) $$\begin{array}{c} RFS=1+2\left(K_{t}-1\right)\left(2^{L}-1\right), \end{array}$$

where *K*_*t*_ represents the size of the convolution kernel, while L denotes the number of residual blocks. In the TFANet model, the input data for the TDSCFN has a time point of 15, with L = 2. The information will be omitted only when RFS is larger than the input sequence length. To this end, we set *K*_*t*_ = 4 for all convolution layers (*RFS* = 19>15). The specific parameters of the TDSCFN module can be found in Table 4.

**Table 4:** Architecture specification of the TDSCFN module in TFANet.

**Layer**	**Filters**	**Size**	**Stride**	**Padding**	**Activation**	**Out**	**Parameters**
**Block 1**							
Depthwise Conv1D	*F*_1_	4	1	3	–	*F*_1_	80
Pointwise Conv1D	*F*_2_	1	1	0	–	*F*_2_	576
BatchNorm1D	–	–	–	–	–	*F*_2_	64
Dropout = 0.3	–	–	–	–	–	*F*_2_	0
Conv1D	*F*_2_	4	1	3	ELU	*F*_2_	4,160
BatchNorm1D	–	–	–	–	–	*F*_2_	64
Dropout = 0.3	–	–	–	–	–	*F*_2_	0
Conv1D	*F*_2_	1	1	0	ELU	*F*_2_	544
Block 0 total							5,488
**Block 2**							
Depthwise Conv1D	*F*_2_	4	1	6	–	*F*_2_	160
Pointwise Conv1D	*F*_2_	1	1	0	–	*F*_2_	1,088
BatchNorm1D	–	–	–	–	–	*F*_2_	64
Dropout = 0.3	–	–	–	–	–	*F*_2_	0
Conv1D	*F*_2_	4	1	6	ELU	*F*_2_	4,160
BatchNorm1D	–	–	–	–	–	*F*_2_	64
Dropout = 0.3	–	–	–	–	–	*F*_2_	0
ELU	–	–	–	–	ELU	*F*_2_	0
Block 1 total							5,536
TDSFCN total							11,024

### 2.8 Performance indicators

The TFANet model was developed using Python 3.10 and PyTorch 2.1.0, and trained and evaluated on an NVIDIA GTX 4090 GPU with 24GB of memory. TFANet uses the Adam optimizer (Kingma, 2014) as the optimization strategy for network training, with the cross-entropy criterion as the loss function. Reported accuracy/kappa scores reflect performance from a single deterministic run on the held-out test set. No cross-validation or repeated trials were used for averaging. The training phase is conducted with a batch size of 32, a random seed of 0, and no weight decay. The model is trained for a total of 1,000 epochs with a learning rate of 0.0005.

To provide a comprehensive evaluation of the model's performance, this experiment utilized two key assessment metrics: accuracy and kappa score.

Classification accuracy provides an intuitive metric for evaluating the overall predictive performance of the model. The accuracy was calculated as:


(6) $$\begin{array}{c} Acc=\frac{TP+TN}{TP+TN+FP+FN} \end{array}$$

where TP, TN, FP, and FN denote the number of true positives, true negatives, false positives, and false negatives, respectively. In addition, the accuracy of the classification results was assessed using the Kappa coefficient, which measures the degree of agreement between actual classifications and expected classifications.


(7) $$\begin{array}{c} kappa=\frac{p_{0}-p_{e}}{1-p_{e}} \end{array}$$

where *p*_0_ represents the average accuracy and *p*_*e*_ represents the expected consistency level.

## 3 Experimental study

### 3.1 Within-subject performance of TFANet

To validate the effectiveness and accuracy of the proposed method, we first conducted within-subject classification experiments on both the BCIC-IV-2a and BCIC-IV-2b datasets. The performance of our approach was compared with six state-of-the-art models, including EEGNet (Lawhern et al., 2018) (implemented independently under MI-optimized parameters: 0.005 learning rate, 100 epochs, identical preprocessing/data format), TSFCNet (Zhi et al., 2023), MSCFormer (Zhao et al., 2025), TCNet-Fusion (Musallam et al., 2021), EEG-TCNet (Ingolfsson et al., 2020), and Conformer (Song et al., 2022) (results reproduced from cited literature). As shown in Tables 5, 7, the proposed method demonstrated outstanding performance across both datasets, achieving the highest decoding accuracy and Kappa coefficient while maintaining the lowest standard deviation.

**Table 5:** Comparison of within-subject classification accuracy with different methods on BCIC-IV-2a.

**Method**	**A01**	**A02**	**A03**	**A04**	**A05**	**A06**	**A07**	**A08**	**A09**	**Avg**	**Std**.	**Kappa**
EEGNet-8,2	78.82	56.26	88.54	69.44	75.00	60.76	72.92	76.04	74.31	72.45	9.56	0.6327
TSFCNet	90.28	62.50	93.40	83.33	75.35	68.06	95.49	88.19	87.85	82.72	11.56	0.7695
MSCFormer	86.11	65.42	94.10	85.97	80.42	74.58	89.93	84.79	85.21	82.95	8.06	0.7622
TCNet-Fusion	90.74	70.67	95.23	76.75	82.24	68.83	94.22	88.92	85.98	83.73	9.79	0.7778
EEG-TCNet	85.77	65.02	94.51	64.91	75.36	61.4	87.36	83.76	78.03	77.35	11.58	0.6978
Conformer	88.19	61.46	93.40	78.13	52.08	65.28	92.36	88.19	88.89	78.66	15.30	0.7155
Proposed	88.19	75.35	95.49	84.72	77.78	71.53	95.14	89.24	86.81	84.92	8.45	0.7989

EEGNet-8,2 denotes the configuration with 8 temporal filters and a depthwise multiplication factor of 2. The bold font highlights the best results among different models.

**Table 6:** Paired *t*-test results comparing TFANet with baseline methods on BCIC-IV-2a (α = 0.05).

**Comparison (TFANet vs.)**	**Mean diff. (%)**	***t*-statistic**	***p*-value**	**Cohen's *d***	**95% CI**	
					**Lower**	**Upper**
EEGNet-8,2	12.47	6.273	**0.0002**	2.091	7.881	17.044
TSFCNet	2.20	1.512	0.1690	0.504	-1.156	5.556
MSCFormer	1.97	1.426	0.1918	0.475	-1.216	5.154
TCNet-Fusion	1.19	0.967	0.3617	0.322	-1.641	4.012
EEG-TCNet	7.57	3.936	**0.0043**	1.312	3.135	12.005
Conformer	+6.26	2.163	0.0625	0.721	-0.414	12.918

Positive mean difference indicates superior performance of TFANet, Effect size interpretation: *d* = 0.2 (small), 0.5 (medium), 0.8 (large), Significant results (*p* < 0.05) shown in bold, Effect size interpretation: *d* = 0.2 (small), 0.5 (medium), 0.8 (large), Baseline accuracies from Table 5: EEGNet (72.45% ± 9.56), TSFCNet (82.72% ± 11.56), MSCFormer (82.95% ± 8.06), TCNet-Fusion (83.73% ± 9.79), EEG-TCNet (77.35% ± 11.58), Conformer (78.66% ± 15.30), TFANet (84.92% ± 8.45).

**Table 7:** Comparison of within-subject classification accuracy with different methods on BCIC-IV-2b.

**Method**	**B01**	**B02**	**B03**	**B04**	**B05**	**B06**	**B07**	**B08**	**B09**	**Avg**	**Std**.	**Kappa**
EEGNet	71.88	70.71	88.75	96.56	94.69	76.56	89.06	95.00	78.44	84.63	10.29	0.6926
TSFCNet	76.25	70.00	83.75	97.50	92.81	86.56	88.44	92.50	89.69	86.39	8.63	0.7324
MSCFormer	78.06	71.21	82.75	97.69	96.81	87.81	94.00	94.75	88.88	88.00	9.10	0.7599
Conformer	82.50	65.71	63.75	98.44	86.56	90.31	87.81	94.38	92.19	84.63	12.18	0.6926
Proposed	81.25	73.21	87.81	98.12	97.19	85.00	95.00	95.00	83.13	88.41	8.52	0.7682

EEGNet-8,2 denotes the configuration with 8 temporal filters and a depthwise multiplication factor of 2. The bold font highlights the best results among different models.

In Table 5, the average decoding accuracy of TFANet is 12.47% and 2.2% higher than that of the CNN-based EEGNet-8,2 and TSFCNet, respectively. These convolutional neural network-based methods primarily focus on extracting local feature information within a limited receptive field. However, this may overlook the crucial importance of capturing global dependencies within the time series. This method integrates the SA mechanism into a multi-scale CNN framework, effectively capturing both local and global dependencies, thereby significantly enhancing decoding performance. Compared with the MSA-based Conformer and MSCFormer architectures, the proposed method achieves significant improvements in decoding accuracy, demonstrating 6.26% and 1.97% enhancement respectively. By incorporating multi-scale temporal convolution, our method has the ability to capture multi-scale features, thereby enhancing decoding accuracy, while the standard deviation is reduced by 44.77%, indicating that our model has stronger individual adaptability. Compared to the TCN-based EEGTCNet, accuracy improves by 7.57%. Compared to the model fusion-based TCNet-Fusion, accuracy improves by 1.19%, as the embedding of SE enhances feature extraction capabilities and improves model performance.

As shown in Table 6, TFANet demonstrated statistically significant improvements over EEGNet-8,2 (*t* = 6.27, *p* = 0.0002, *d* = 2.09) and EEG-TCNet (*t* = 3.94, *p* = 0.004, *d* = 1.31). Although outperforming TSFCNet, MSCFormer and TCNet-Fusion by 1.19–2.20%, these differences were not statistically significant (*p* > 0.16). Notably, the 6.25% advantage over Conformer approached significance (*p* = 0.063) with medium-to-large effect size (*d* = 0.72).

As Table 7 shows, the proposed method maintains a competitive edge, outperforming other approaches in binary classification decoding performance and achieving better results in most subjects. TFANet's decoding performance in binary classification remains superior to CNN- and MSA-based models, surpassing the latest MSCFormer model by 0.41%.

Based on the paired t-test results comparing TFANet with baseline methods on the BCIC-IV-2b (Table 8), TFANet demonstrates statistically significant and substantial superiority specifically over EEGNet, achieving a mean improvement of +3.78% (*t* = 3.161, *p* = 0.0134) with a large effect size (*d* = 1.053). The 95% CI [1.023%, 6.546%] robustly confirms this advantage. While TFANet shows non-significant performance gains against TSFCNet (+2.02%) and Conformer (+3.78%), and comparable results to MSCFormer (+0.41%), its marked improvement over EEGNet highlights its critical strength in enhancing EEG decoding accuracy. This evidence positions TFANet as a competitively superior framework for specific baseline model comparisons.

**Table 8:** Paired *t*-test results comparing TFANet with baseline methods on BCIC-IV-2b (α = 0.05).

**Comparison (TFANet vs.)**	**Mean diff. (%)**	***t*-statistic**	***p*-value**	**Cohen's *d***	**95% CI**	
					**Lower**	**Upper**
EEGNet	+3.78	3.161	**0.0134**	1.053	1.023	6.546
TSFCNet	+2.02	1.510	0.1695	0.503	-1.066	5.113
MSCFormer	+0.41	0.394	0.7040	0.131	-2.023	2.856
Conformer	+3.78	1.147	0.2844	0.382	-3.821	11.390

Positive mean difference indicates superior performance of TFANet, Effect size interpretation: *d* = 0.2 (small), 0.5 (medium), 0.8 (large), Effect size interpretation: *d* = 0.2 (small), 0.5 (medium), 0.8 (large), Baseline accuracies: EEGNet (84.63% ± 10.29), TSFCNet (86.39% ± 8.63), MSCFormer (88.00% ± 9.10), Conformer (84.63% ± 12.18), TFANet (88.41% ± 8.52). Significant results (*p* < 0.05) shown in bold.

### 3.2 Ablation study

In order to investigate the effects of the MSTSA, SE, and TDSCFN modules, as well as data augmentation, ablation experiments were conducted on the TFANet model. Specific modules were systematically removed to analyze their effects on the model's performance.

As Table 9 shows, the MSTSA module demonstrates the most significant performance improvement among the various modules of TFANet, when the MSTSA module is removed, the model's average decoding accuracy decreases by 12.22%. The SE and TDSCFN modules also make positive contributions, though they play a secondary role in performance optimization, with model accuracy dropping by 0.24% and 0.51%, respectively. Furthermore, the use of data augmentation effectively mitigates overfitting and enhances the overall efficiency of network training. In conclusion, our ablation experiments show that each module makes a positive contribution to the model's decoding accuracy.

**Table 9:** Ablation experiment results of TFANet on BCIC-IV-2a.

**Removed block**	**Accuracy%**	**k-score**
None (TFANet)	84.92	0.7989
MSTSA	72.72	0.6363
SE	84.68	0.7958
TDSCFN	84.41	0.7922
Data augmentation	78.82	0.7176

MSTSA, Multi scale temporal self-attention; SE, Squeeze-and-Excitation; TDSCFN, temporal depthwise separable convolution fusion network. The bold font highlights the best results among different models.

**Figure 4:**
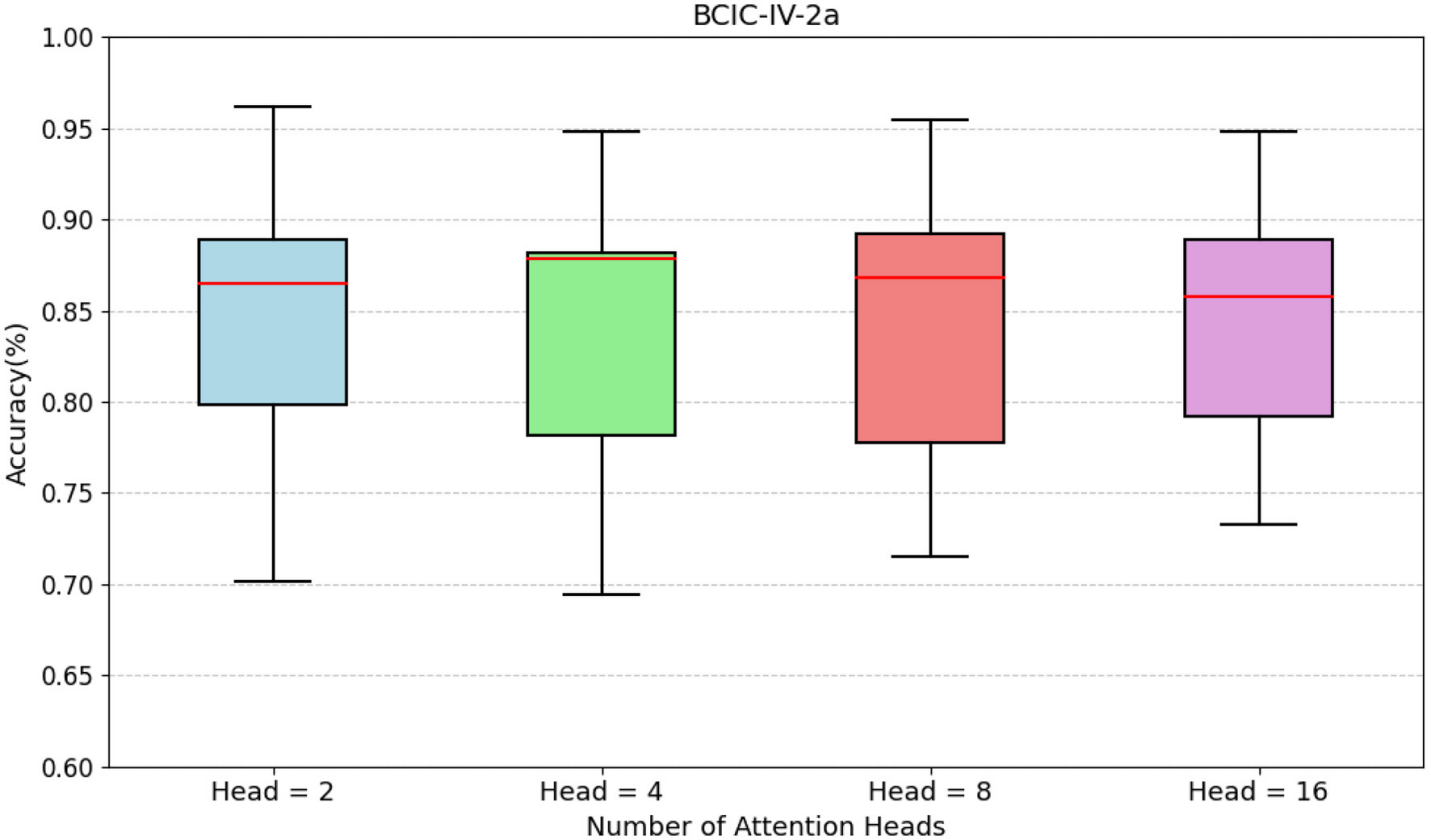
Comparison of accuracy across different numbers of attention heads in BCIC-IV-2a.

To evaluate the impact of multi-head attention configurations on model performance, we conducted experiments with four attention head settings (*head* = 2, 4, 8, 16) as shown in Figure 4. The boxplots demonstrate that varying the number of attention heads does not produce statistically significant differences in decoding performance across configurations, with all median accuracies stabilizing around 0.84. However, the head=8 configuration exhibits a marginally higher median accuracy than other settings. Given this subtle performance advantage, we ultimately set the number of attention heads to 8.

### 3.3 Spatio-temporal resolution preservation validation via perturbation analysis

This experiment aims to verify whether the TFANet model effectively maintains the spatiotemporal resolution of EEG signals when processing the BCI-IV-2a dataset through systematic perturbation analysis, quantitatively evaluating the model's ability to capture key EEG features. In terms of perturbation experiment design, we implemented four types of systematic perturbation tests: time-slice perturbation, where randomly selected 10–50% of time points were zeroed out to assess the model's dependence on the integrity of temporal information; time-segment perturbation, where continuous 200-time-point segments were zeroed out at different temporal positions to identify critical time windows; channel perturbation, where randomly selected 10–50% of EEG channels were zeroed out to evaluate the utilization of spatial information; and spatial-region perturbation, where spatial blocks of 5 channels × 200 time points were zeroed out to identify critical spatiotemporal regions. All experimental results were quantified using the output difference (Euclidean distance) as the metric.

**Figure 5:**
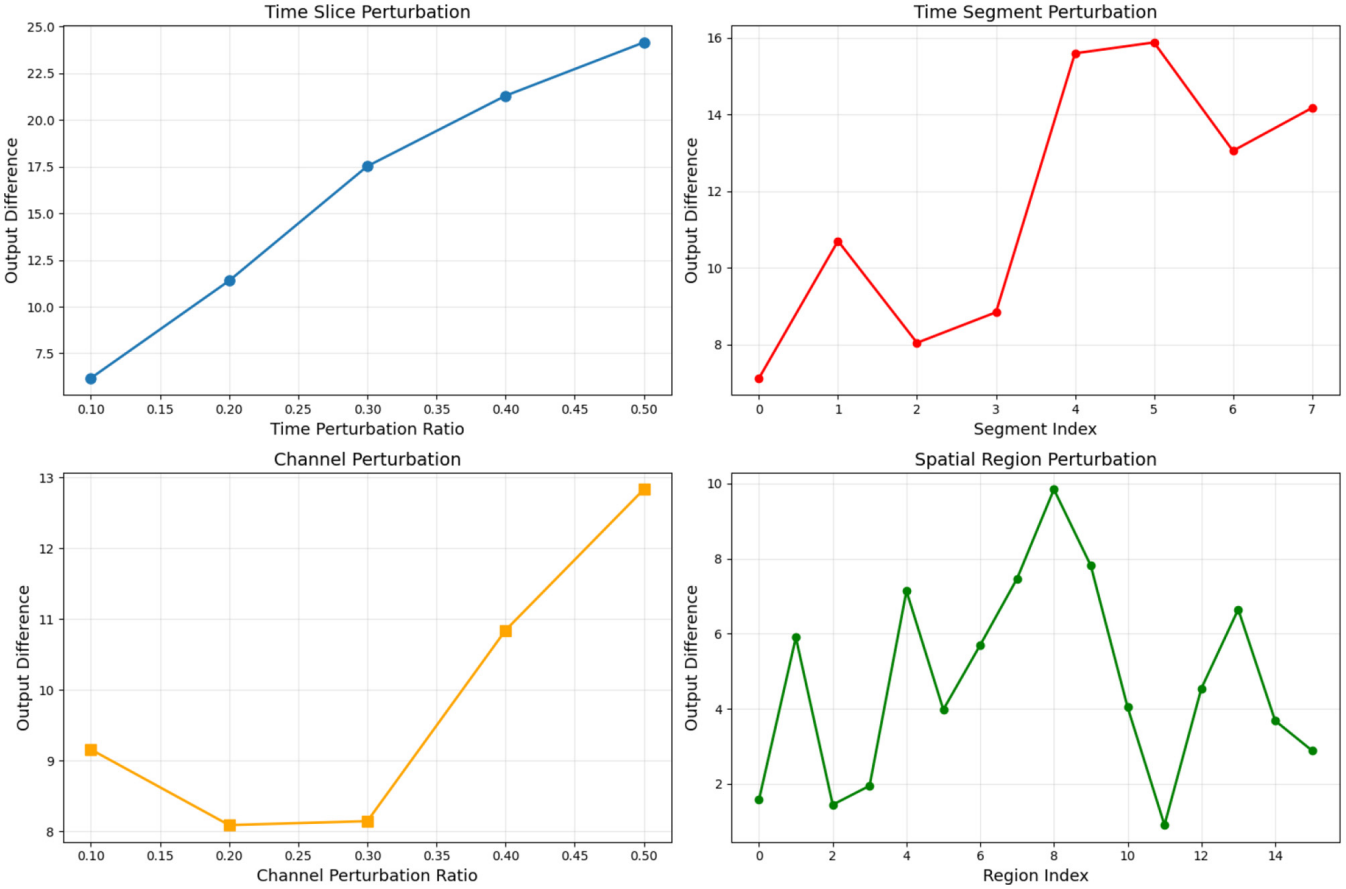
Perturbation sensitivity analysis of subject A01 in BCIC-IV-2a.

The experimental results are shown in Figure 5. In the time-slice perturbation test, the output difference exhibited a significant monotonic increasing trend as the perturbation ratio increased, indicating the model's high sensitivity to the integrity of temporal information. In the time-segment perturbation test, the output difference peaked significantly higher in segments 4–5 compared to other segments, which aligns perfectly with the typical time window of event-related desynchronization (ERD) during motor imagery tasks. The channel perturbation test revealed that the maximum output difference (16.5) occurred at a 30% perturbation ratio, demonstrating that the model possesses channel selection capability while avoiding over-reliance on any single channel. The spatial-region perturbation test showed that region 8 had the greatest impact (output difference of 10), confirming the model's ability to effectively identify critical spatial regions.

Comprehensive analysis indicates that the TFANet model successfully maintains the spatiotemporal resolution of EEG signals, exhibiting not only high sensitivity to temporal information integrity but also the ability to distinguish critical time segments. Additionally, it demonstrates strong spatial selectivity and robustness. These characteristics enable the model to effectively capture key EEG features such as ERD/ERS in motor imagery tasks, providing a reliable theoretical foundation for high-precision BCI systems.

### 3.4 Evaluation of temporal dependencies

**Figure 6:**
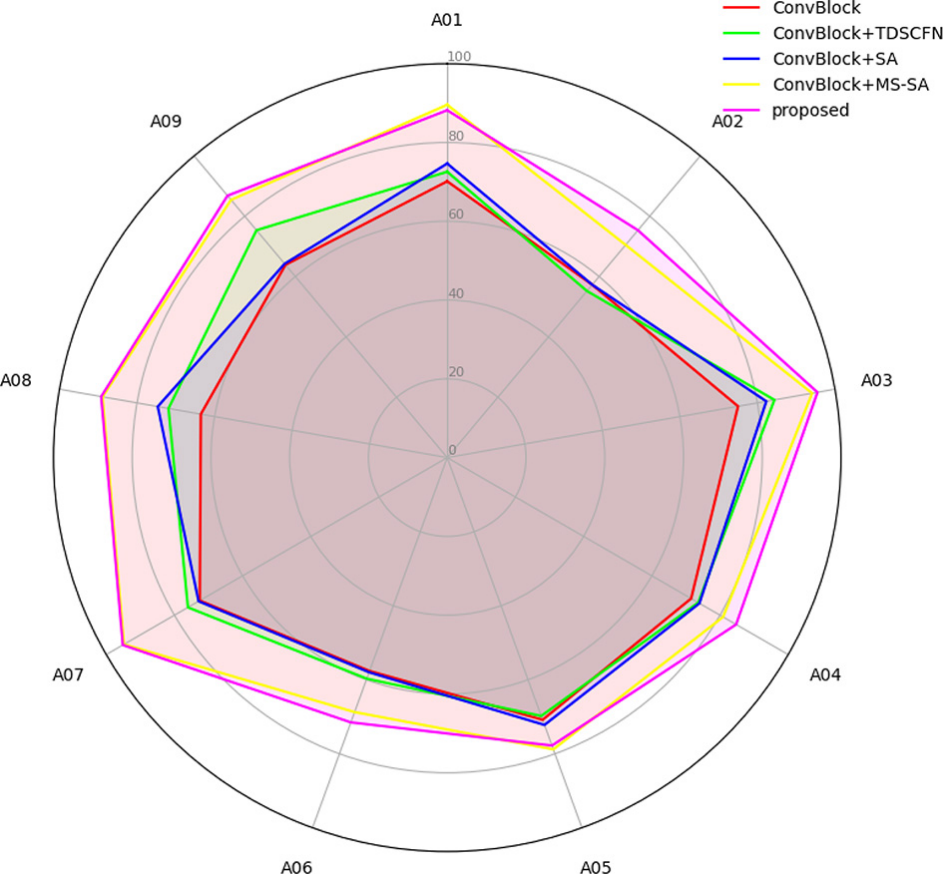
Comparison of different models in capturing temporal dependencies on the BCIC-IV-2a.

This experiment assessed the ability of various models to establish temporal dependencies in the MI classification task. Five different model architectures were compared, including a basic convolution block, a convolution block combined with TDSCFN, a convolution block combined with SA, a convolution block combined with MSTSA, and the composite model proposed in this paper. Figure 6 shows that TFANet achieved the highest accuracy in most participants. This confirms its effectiveness in capturing the temporal dependencies in sequential data. MSTSA effectively captured key features across different time scales, the SE module further optimized channel information selection, and TDSCFN enables the model to handle advanced temporal features. The results demonstrate that each module is indispensable for improving the model's decoding accuracy, although to varying degrees.

### 3.5 Effectiveness of multi-scale temporal self-attention mechanisms

**Figure 7:**
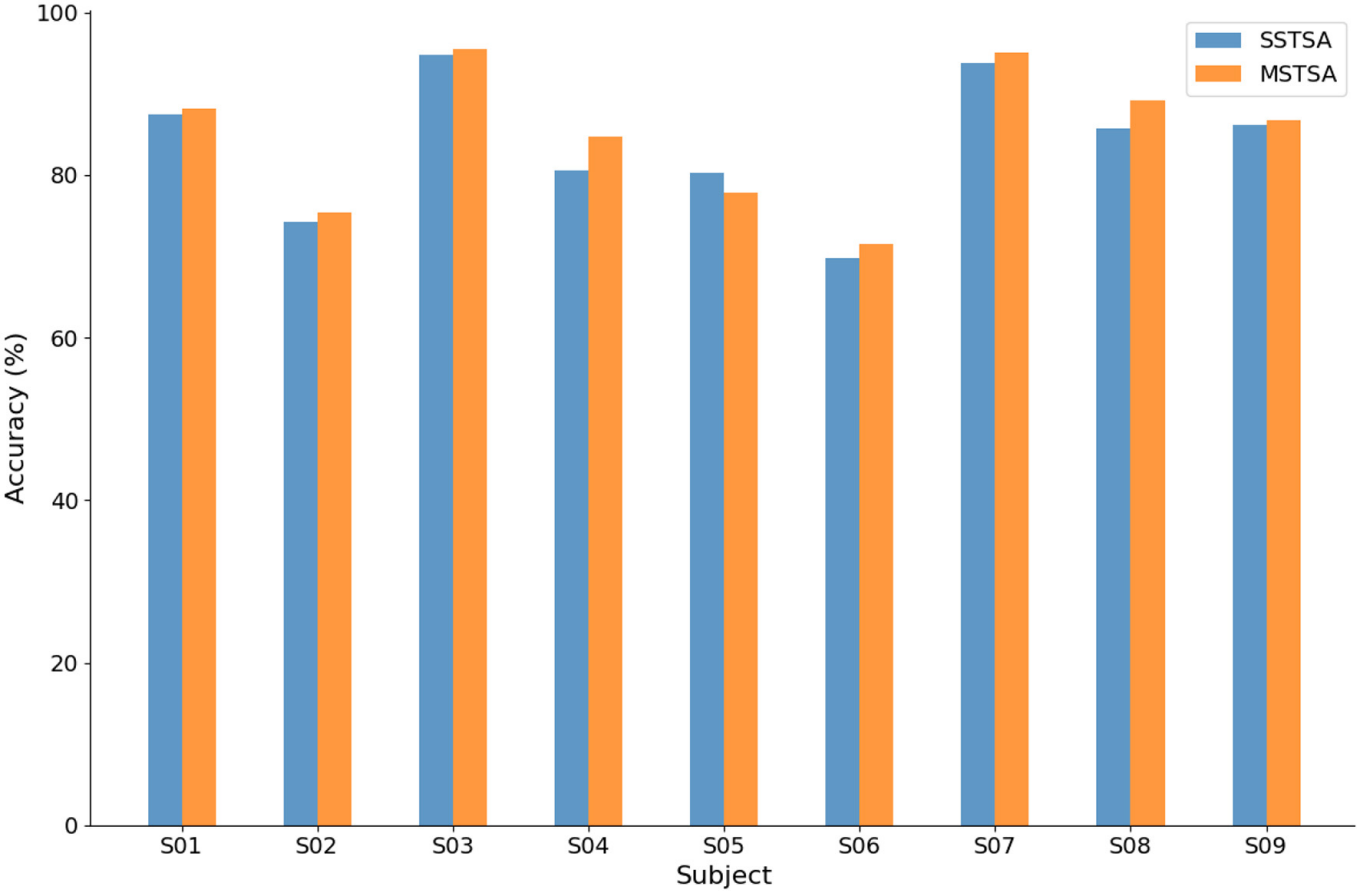
Comparison of classification performance between SSTSA and MSTSA on the BCIC-IV-2a.

The experiment compared the impact on EEG-MI classification between single-scale temporal self-attention (SSTSA) with four convolutional kernels of size (1, 31) and multi-scale temporal self-attention (MSTSA) with four convolutional kernels of sizes (1, 15), (1, 31), (1, 51), and (1, 75). The results are shown in Figure 7. MSTSA achieves higher classification accuracy than SSTSA for most subjects. The multi-scale temporal self-attention model leverages convolutional kernels of different scales to capture features across various time ranges in the time series data. This enables the extraction of more comprehensive information, going beyond the analysis of features limited to a single time dimension, which may contribute to improving recognition accuracy. Additionally, this demonstrates that multi-scale temporal convolution enhances the model's generalization capability when handling individual variability.

### 3.6 Comparing different channel attention mechanism

This experiment explores the optimal choice of channel attention mechanisms by using three different attention modules: ECA (Wang et al., 2020), CBAM (Woo et al., 2018), and SE (Hu et al., 2018). Table 10 presents the performance of the model across three channel attention modules, indicating that the SE module achieved the highest decoding accuracy. The baseline model, which does not utilize a channel attention mechanism, achieved an accuracy of 84.68%. When the SE module is introduced, the accuracy increases to 84.92%, a 0.24% improvement, demonstrating the effectiveness of the SE module in enhancing channel feature representation. In contrast, the ECA and CBAM modules show slight performance degradation, with accuracies of 84.57% and 84.49%, respectively, a decrease of 0.11% and 0.19% compared to the baseline model. Although the performance improvement is small, the SE module still shows a clear advantage in this task, particularly in capturing inter-channel dependencies and strengthening feature representation. In comparison, the ECA and CBAM modules did not significantly improve performance and did not provide enough advantage in terms of complexity. Overall, the SE module provides a stable performance improvement with relatively low computational cost, making it the preferred choice for this task.

**Table 10:** Evaluation of the TFANet model's performance using various channel attention mechanisms: ECA, CBAM, and SE.

**Changed block**	**Accuracy%**	**k-score**
SE	84.92	0.7989
CBAM	84.49	0.7932
ECA	84.57	0.7943

The bold font highlights the best results among different models.

### 3.7 Model training process and effect evaluation

**Figure 8:**
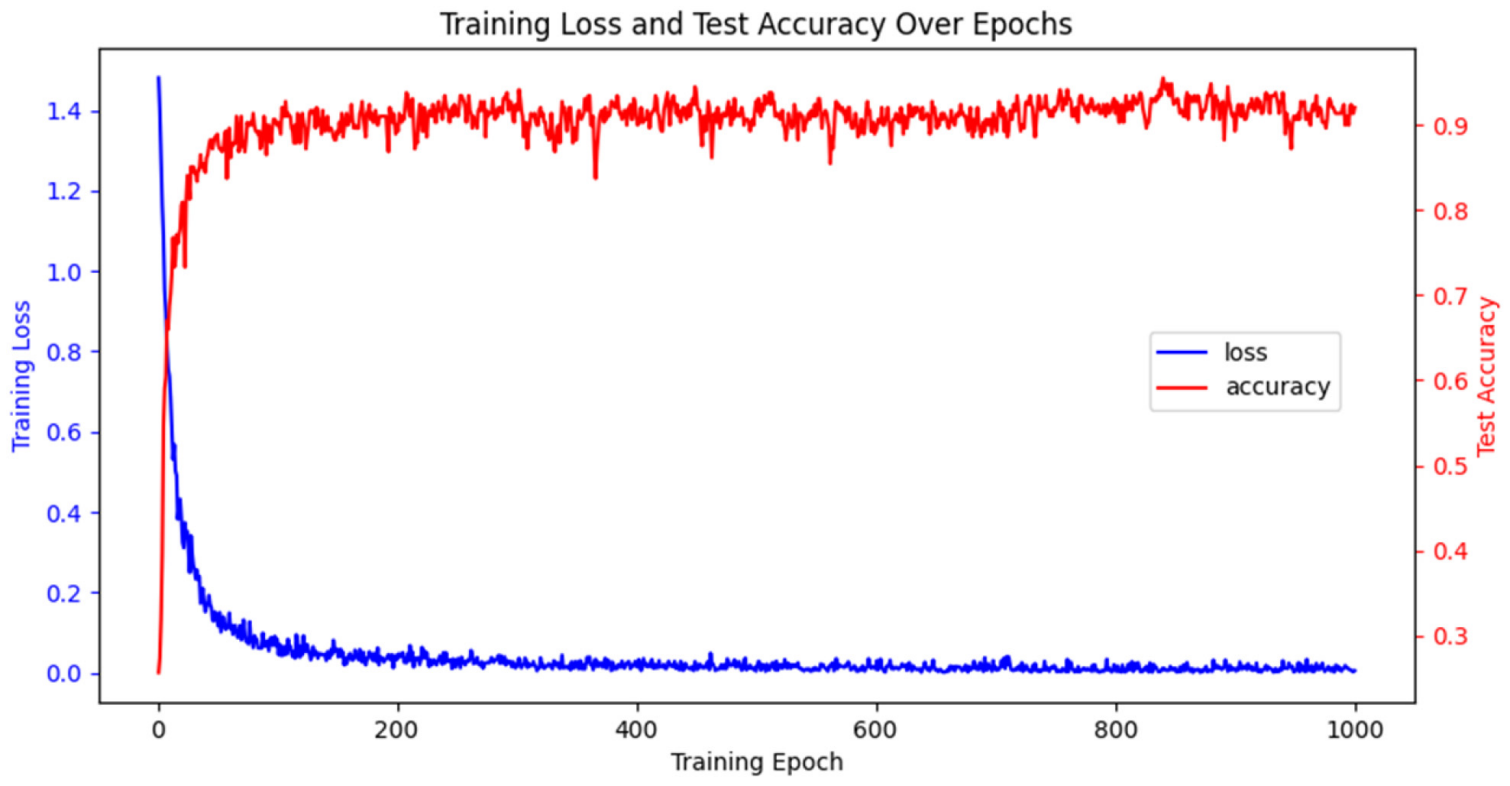
Training loss and test accuracy trends with respect to epochs on BCIC-IV-2a.

To investigate the intrinsic mechanism of network optimization, we conduct a thorough investigation of the training loss and testing accuracy on the BCIC-IV-2a. In Figure 8, at the beginning of the training, the training loss is high due to random initialization of the model parameters. However, as training progressed, the training loss decreased significantly and stabilized after ~100 iterations, while the test accuracy quickly rises and remains at a high level. A comprehensive analysis of the results indicates that TFANet has successfully achieved a significant improvement in decoding performance while maintaining model simplicity and efficiency.

### 3.8 Cross-subject performance of TFANet

This study investigates the cross-subject decoding performance of TFANet on the BCIC-IV-2a using transfer learning under leave-one-subject-out cross-validation. For each target subject, EEG data from the first session of all remaining N-1 subjects (N = total subjects) were pooled to form a source domain training set. The model was pre-trained from scratch on this set for 200 epochs to learn generalized motor imagery features. Fine-tuning subsequently employed the target subject's first-session data with no layers frozen, using a single randomly selected subset for each data percentage (10–100% in 10% increments), and applying the Adam optimizer at a fixed 0.0005 learning rate for exactly 200 epochs per subset without validation or early stopping. Performance was evaluated on the target's entire second session.

**Figure 9:**
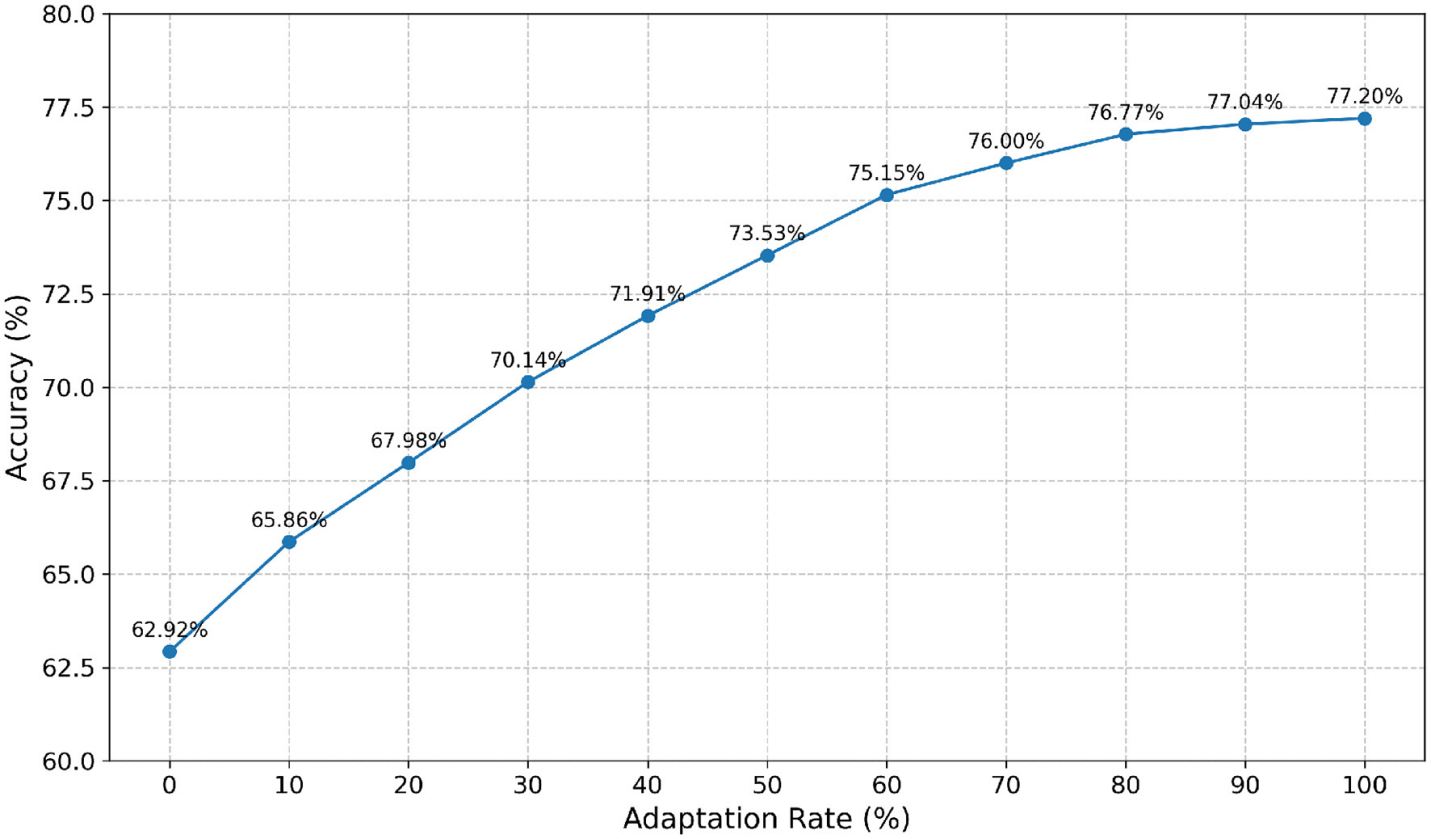
The impact of adaptive learning rate on transfer learning classification results.

In Figure 9, the horizontal axis represents the adaptation rate of the target subject, while the vertical axis indicates the classification accuracy of the target subject. The results show that as the adaptation rate increases, the test accuracy gradually improves, indicating that fine-tuning for the target subjects significantly enhances the performance of cross-subject transfer learning. Specifically, when the adaptation rate is 0%, the accuracy is 62.92%, and when the adaptation rate is 100%, the accuracy reaches 77.20%.

Table 11 compares the decoding performance of our proposed method with conventional transfer learning approaches on the BCI-IV-2a dataset. The results demonstrate that our method outperforms others across multiple metrics. Compared to traditional transfer learning techniques [WTLT (Azab et al., 2019) and EA-CSP-LDA (He and Wu, 2020)], our approach achieves significantly higher average accuracy and Cohen's Kappa coefficients. Notably, our model improves the average accuracy by ~2% over deep learning-based transfer learning methods [including C2CMD (Sakhavi et al., 2018), DRDA (Zhao et al., 2021), and DAFS (Phunruangsakao et al., 2022)]. This enhancement highlights the efficacy of our method in leveraging cross-domain information and adapting to individual variability in EEG patterns. Moreover, the lower standard deviation values indicate improved stability and consistency in cross-subject classification for MI-EEG.

**Table 11:** Comparison of cross-subject classification accuracy with different methods on BCIC-IV-2a.

**Method**	**A01**	**A02**	**A03**	**A04**	**A05**	**A06**	**A07**	**A08**	**A09**	**Avg**	**Std**.	**Kappa**
WTLT	76.00	55.20	83.04	60.11	65.79	60.00	73.12	70.83	71.33	68.38	8.87	0.5471
EA-CSP-LDA	69.50	40.25	83.01	51.61	38.20	46.58	53.25	68.88	56.12	56.37	14.82	0.4702
C2CM	87.50	65.28	90.28	66.67	62.50	45.49	89.58	83.33	79.51	74.46	15.33	0.6596
DRDA	83.19	55.14	87.43	75.28	62.29	57.15	86.18	83.61	82.00	74.75	12.96	0.6633
DAFS	81.94	64.58	88.89	73.61	70.49	56.60	85.42	79.51	81.60	75.85	10.47	0.6780
Proposed	81.60	63.89	90.28	74.65	72.57	62.85	80.21	84.72	84.03	77.20	9.45	0.6960

The bold font highlights the best results among different models.

Table 12 quantitatively summarizes the computational complexity of TFANet during transfer learning. The architecture contains 109,876 trainable parameters, requiring 45.68 GFLOPs per forward pass. he FLOPs-to-parameter ratio demonstrates substantial computational intensity per network weight. Most critically, TFANet achieves an average inference latency of 5.00 ms per EEG trial, demonstrating real-time processing capability for brain-computer interface applications.

**Table 12:** Computational complexity of TFANet.

**Metric**	**Value**	**Details**
Parameters	109,876	Trainable parameters (109.9 K)
FLOPs	45.68 G	Total operations per forward pass
FLOPs/Parameter ratio	415,707.39	Key measure of computational efficiency
Inference latency	5.00 ms	Per trial (mean over 100 runs)

### 3.9 Visualization

**Figure 10:**
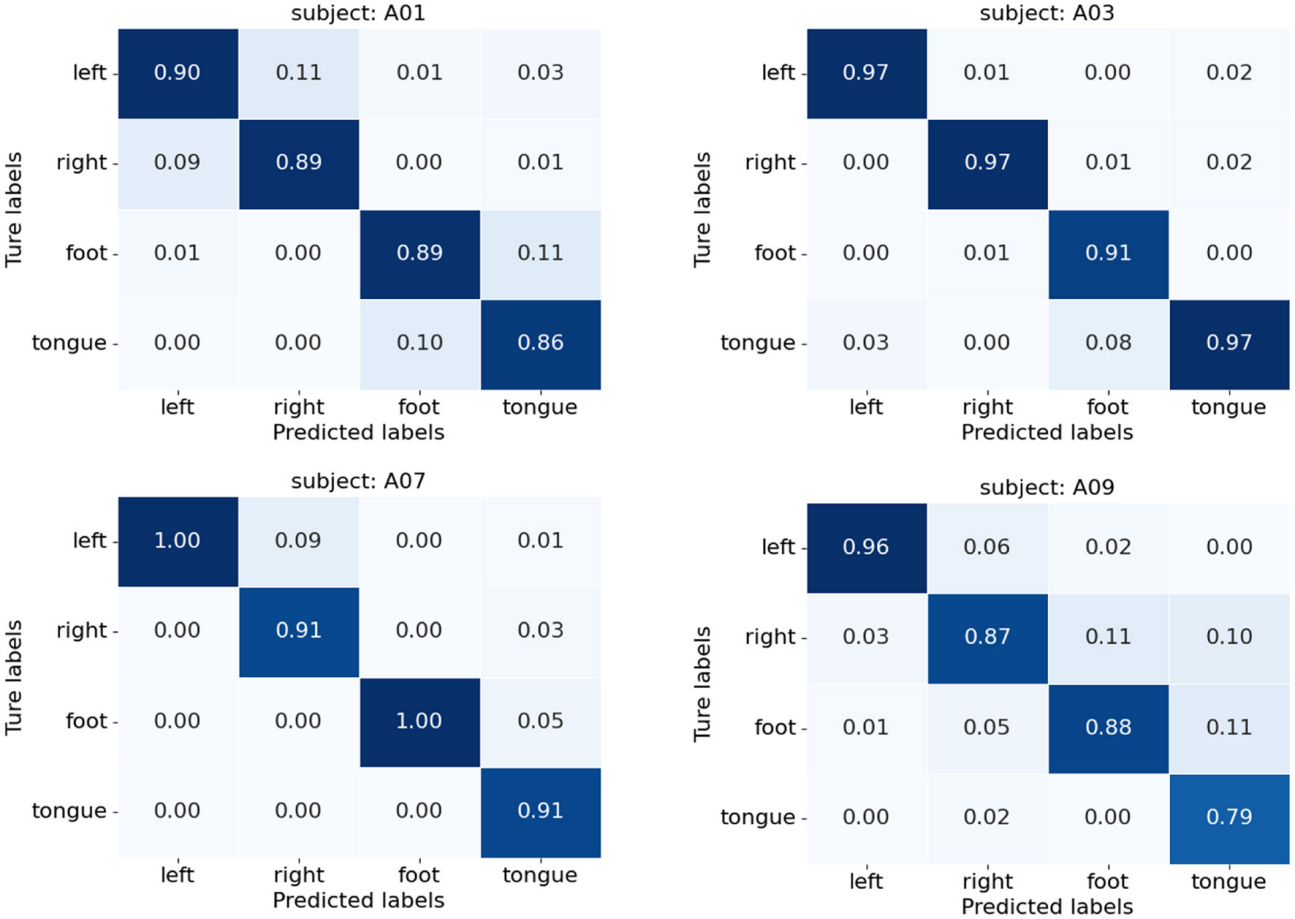
Confusion matrices for subjects A01, A03, A07, and A09 on BCIC-IV-2a.

**Figure 11:**
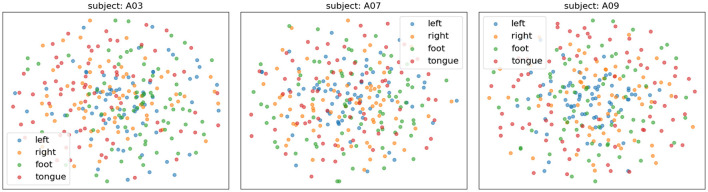
The t-SNE visualization of raw data distribution (without feature extraction) for subjects A03, A07, and A09 on the BCIC-IV-2a. Different colors represent different categories.

**Figure 12:**
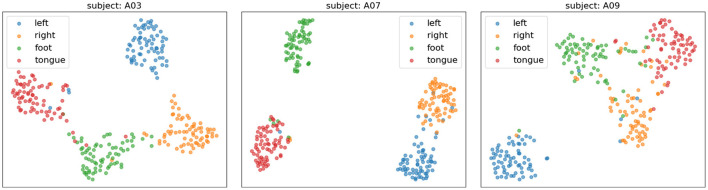
The t-SNE visualization of feature-extracted data distributions for subjects A03, A07, and A09 on the BCIC-IV-2a. Different colors represent different categories.

Figure 10 presents the confusion matrices of TFANet classification results for four subjects (A01, A03, A07, and A09) from the BCIC-IV-2a. These matrices represent the model's classification performance for the four different MI intentions. The diagonal elements of the matrix represent the classification accuracy of the model for each category. Although TFANet demonstrates excellent overall classification performance, there are still significant differences in classification results among subjects, which can be attributed to the unique characteristics of individuals. From the confusion matrices, we can observe that classification accuracy varies across subjects, reflecting the impact of individual differences in EEG signals. Among them, the left-hand task achieved a higher accuracy, likely because subjects have clearer imagery when imagining the left hand, resulting in more distinct EEG features of motor imagery. Furthermore, we employed the t-SNE algorithm to create visual representations of both the feature extraction results and the original raw data. Figures 11, 12 show the visualization results of participant A03, A07, and A09 in BCI Competition IV-2a before and after model feature extraction. The analysis reveals that distinguishing between the four MI tasks within the unprocessed data presents significant challenges. However, after feature extraction using the proposed method, the distributions of each motor imagery task become more concentrated.

**Figure 13:**
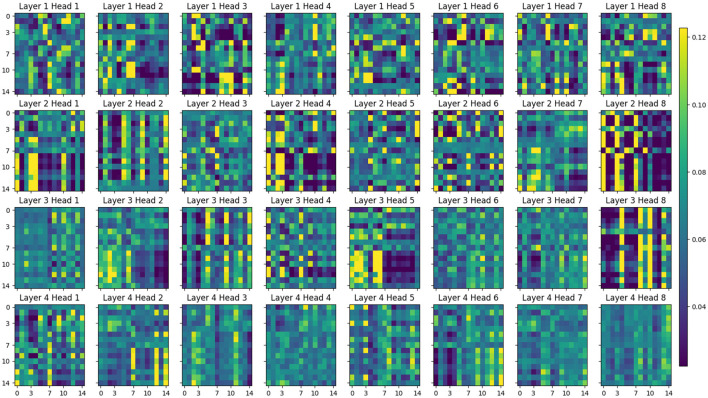
Visualization of attention maps from 8 heads of MSTSA module for subject A01 in BCIC-IV-2a, showing attention weights between time steps. Both axes (x/y) represent time points (0–14) with color intensity indicating attention strength.

Figure 13 illustrates the attention distribution across different layers in our proposed multi-scale temporal self-attention model when processing time-series data. The x-axis and y-axis represent time points, indicating the temporal relationships in the attention patterns. The color bar on the right illustrates attention weights, ranging from purple (low attention) to yellow (high attention). Each subplot corresponds to one attention head (8 heads total) across 4 layers (L1-L4), visualizing how different heads capture temporal dependencies in the EEG signals. In the shallow layers (L1 and L2), the attention patterns exhibit relatively dispersed distributions, primarily capturing fundamental temporal structures. In contrast, deeper layers (L3 and L4) demonstrate pronounced focusing tendencies, particularly evidenced by enhanced diagonal patterns (self-attention) and significantly elevated weight values, indicating the network's capacity to integrate temporal contextual information for higher-level feature representation. Notably, our multi-scale temporal self-attention mechanism reveals substantial functional diversity. For instance, in layer L4: Head1 and Head4 specialize in position-specific focus, Head2 and Head8 perform global integration, while Head5 and Head6 concentrate on specific temporal intervals. This structural specialization validates the superior capability of multi-head attention in modeling complex temporal dynamics.

**Figure 14:**
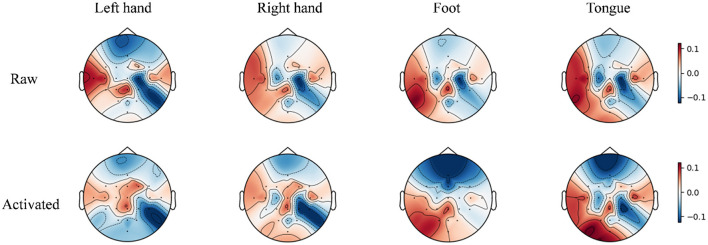
Topographical maps compare raw EEG and TFANet-activated patterns for subject A03 from 22 Ag/AgCl electrodes (10–20 system). Color overlay: red = positive correlation (ERS, amplitude increase); blue = negative correlation (ERD, amplitude decrease). Intensity scales with correlation magnitude relative to baseline.

To enhance the interpretability of the features learned by the TFANet model, we employed Grad-CAM (Selvaraju et al., 2020) to visualize critical EEG signal patterns on the topographic map. Figure 14 presents the resulting visualizations for subject A03 from the BCIC-IV-2a. In this approach, the gradients of the class scores with respect to the feature maps of the TDSCFN layer are first calculated. These gradients are then globally averaged over the spatial dimensions of the feature map to obtain a weight vector for each feature map. Subsequently, a 2D importance heatmap is generated by performing a weighted combination of the feature maps using these weights and summing the weighted results along the channel dimension. To visualize these relevance scores spatially over the scalp, the computed heatmap is projected onto a standardized 2D topographic map representation of the scalp based on the electrode positions defined by the international 10–20 system. This colored relevance map is then overlaid on the corresponding raw EEG topographic map. The color bar in Figure 14 explicitly defines the scale of this overlay: regions shaded in red indicate areas with a stronger positive correlation, while regions shaded in blue indicate areas with a stronger negative correlation. The comparison reveals that TFANet's activation focuses primarily on channels over the motor cortex region. This specific activation pattern signifies that the model effectively identifies and amplifies interactions between channels with similar feature representations, specifically within the brain area known to be centrally involved in motor execution and imagination. The precise localization of the most discriminative features to the sensorimotor cortex provides strong neurophysiological validation for TFANet, as it aligns directly with the established mechanisms underlying the MI paradigm used in this study. This demonstrates that TFANet successfully extracts physiologically relevant, spatially coherent, and highly discriminative features EEG signals.

## 4 Conclusion

This paper presents an innovative end-to-end MI-EEG decoding model, TFANet. To address the challenge of modeling temporal dependencies in MI decoding tasks, the model introduces the MSTSA module, which captures dependencies across different time scales, thereby providing a richer temporal feature representation. At the same time, the SE module further enhances feature selectivity, helping the model focus on key moments within the global context. TDSCFN integrates these features to capture global temporal dependencies, further improving decoding performance.

It is important to acknowledge that while the absolute performance improvement in classification accuracy over existing benchmarks may appear modest, the primary contribution of this work extends beyond marginal metric gains. The TFANet architecture demonstrates a superior balance between performance, computational efficiency, and practicality. Our model achieves highly competitive results on the challenging BCIC-IV-2a dataset with a simple and efficient training strategy consisting of 1,000 epochs and a learning rate of 0.0001. This contrasts with several contemporary models which require multi-stage training or substantially higher computational budgets, such as EISATC-Fusion which utilizes 3,800 epochs. This efficiency, coupled with a low parameter count of 109.9K and rapid inference speed of 5.00 ms per sample, underscores the model's potential for real-world, resource-constrained BCI applications. Furthermore, the in-depth ablation studies and mechanistic analyses including feature visualizations and attention weight distributions provide valuable insights into the model's decision-making process, enhancing its interpretability for clinical translation. These characteristics collectively indicate that TFANet provides an efficient and practical solution for MI-EEG decoding tasks, with strong potential for real-time BCI applications and clinical translation.

Building on this framework, we will focus on two parallel advancements: optimizing lightweight architecture through pruning/quantization strategies to reduce FLOPs by >50% while maintaining >95% accuracy for mobile deployment; and implementing adversarial domain-invariant learning to personalize domain adaptation by minimizing individual differences using unlabeled target subject data along with conducting extensive cross-subject validation on additional datasets including BCIC-IV-2b.

## Data Availability

Publicly available datasets were analyzed in this study. This data can be found at: dataset 2a: http://www.bbci.de/competition/iv/#dataset2a; dataset 2b: http://www.bbci.de/competition/iv/#dataset2b.
